# Comparative Transcript Profiling of a Male Sterile Cybrid Pummelo and Its Fertile Type Revealed Altered Gene Expression Related to Flower Development

**DOI:** 10.1371/journal.pone.0043758

**Published:** 2012-08-28

**Authors:** Bei-Bei Zheng, Xiao-Meng Wu, Xiao-Xia Ge, Xiu-Xin Deng, Jude W. Grosser, Wen-Wu Guo

**Affiliations:** 1 Key Laboratory of Horticultural Plant Biology (Ministry of Education); National Key Laboratory of Crop Genetic Improvement, Huazhong Agricultural University, Wuhan, People’s Republic of China; 2 Citrus Research and Education Center, University of Florida, Lake Alfred, Florida, United States of America; United States Department of Agriculture, United States of America

## Abstract

Male sterile and seedless characters are highly desired for citrus cultivar improvement. In our breeding program, a male sterile cybrid pummelo, which could be considered as a variant of male fertile pummelo, was produced by protoplast fusion. Herein, ecotopic stamen primordia initiation and development were detected in this male sterile cybrid pummelo. Histological studies revealed that the cybrid showed reduced petal development in size and width, and retarded stamen primordia development. Additionally, disorganized cell proliferation was also detected in stamen-like structures (fused to petals and/or carpel). To gain new insight into the underlying mechanism, we compared, by RNA-Seq analysis, the nuclear gene expression profiles of floral buds of the cybrid with that of fertile pummelo. Gene expression profiles which identified a large number of differentially expressed genes (DEGs) between the two lines were captured at both petal primordia and stamen primordia distinguishable stages. For example, nuclear genes involved in nucleic acid binding and response to hormone synthesis and metabolism, genes required for floral bud identification and expressed in particular floral whorls. Furthermore, in accordance with flower morphology of the cybrid, expression of *PISTILLATA* (*PI*) was reduced in stamen-like structures, even though it was restricted to correct floral whorls. Down-regulated expression of *APETALA3* (*AP3*) coincided with that of *PI*. These finding indicated that, due to their whorl specific effects in flower development, citrus class-B MADS-box genes likely constituted ‘perfect targets’ for CMS retrograde signaling, and that dysfunctional mitochondria seemed to cause male sterile phenotype in the cybrid pummelo.

## Introduction

In the past two decades, somatic hybridization have been a powerful tool in citrus breeding for specific traits such as seedlessness and disease resistance, and numerous somatic hybrids have been produced for both scion and rootstock improvement [1], [2], [3], [4], [5]. During symmetric protoplast fusion and regeneration process in citrus, diploid cybrids are often generated from somatic fusion of embryogenic culture-derived protoplasts of one diploid parent with leaf-derived protoplasts of a second diploid parent [6]. Recovered cybrids are generally composed of the nuclear genome of the leaf parent, the mitochondrial genome of the embryogenic callus/suspension parent, and a randomly inherited chloroplast genome [7], [8]. Considering the possibility of targeted mitochondrial genome transfer and the importance of seedless trait for fresh citrus market, we put forward a strategy to transfer male sterile and seedless trait by somatic cybridization in efforts to reduce the seed content of important cultivars [9]. In our previous study, diploid cybrids were generated [10] by transferring the male sterile cytoplasm of Satsuma mandarin (*Citrus unshiu* Marc.), a seedless variety with CMS character [11], to seedy citrus cultivars. An interesting diploid cybrid (G1+HBP) was regenerated with the nuclear genome and chloroplast DNA from the leaf parent Hirado Buntan pummelo (*C. grandis* (L.) Osbeck) (HBP, a seedy variety with attractive fruit quality) and mitochondrial genome from the callus parent Satsuma mandarin cv. Guoqing No. 1 (G1). In other words, male sterile cybrid pummelo could be considered as a pummelo variant with foreign mitochondria, but maintaining pummelo cultivar integrity. With this strategy, other citrus cybrids and somatic hybrids had also been created [3], [12], [13]. The production of cybrids was a valuable method for improvement of citrus seedless breeding and providing ideal materials for understanding the CMS trait.

CMS, resulted from disturbances in nuclear-mitochondrial interaction, was a failure to initiate stamen development and produce functional pollen [14], [15], [16]. Mitochondrial genes that determined CMS could be suppressed or counteracted by nuclear genes. Alterations in the mitochondrial DNA or mitochondrial gene expression also resulted in changed expression of certain nuclear genes [14], [16], [17]. Focusing on the CMS trait provided a convenient way to probe the role of the mitochondria in reproductive development and gain new insight into interactions between the mitochondrial and nuclear genomes [14], [15]. It was widely accepted that CMS was associated with mitochondrial genome rearrangement, and many mitochondrial genes that determined the trait could be suppressed or activated by nuclear restored genes [16], [18]. In addition to naturally occurring, CMS could be artificially synthesized by either sexual crossing or protoplast fusion with the mitochondrial genome of one species moved into the nuclear background of another. The novel nuclear-cytoplasm combination of CMS lines resulted in aberrant expression of mitochondrial gene, and different CMS phenotypes had been attributed to the generation of chimerical open reading frames [18], [19], [20]. Previous studies also showed the phenomena that mitochondrial genome of the CMS line strongly influenced expression profiles of nuclear genes, which indicated the importance of retrograde signaling between mitochondria and nucleus [19], [21]. However, further inquiry is still needed to determine how mitochondrial DNA can regulate the expression levels of nuclear genes involved in male sterility.

Because of nucleus-mitochondria incompatibility, CMS lines exhibited developmental defects in male gametophyte and/or floral organs. CMS could occur at different stages during floral meristem identification and development. In addition to failed pollen production, CMS lines were accompanied by abnormal floral morphology, such as homeotic conversions of floral organ. Here, we report the first woody perennial somatic cybrid line (G1+HBP) with male sterile characteristics in citrus. The morphology of floral buds in G1+HBP was similar to that of leaf parent HBP during early stages of floral bud development; however, phenotypic differences were distinguishable at stamen primordia stages, as flowers developed with retarded development of stamen primordia and few distorted stamen-like structures fused to petals/carpel, with reduced petals in size and width. Similar abnormal flower phenotypes (such as homeotic conversions of the third-whorl organs) had been widely reported in CMS lines of herbaceous plant species, such as *Nicotiana tabacum*, *Triticum aestivum*, *Daucus carota*, and *Brassica napus* [22], [23], [24], [25]. Morphology changes of floral buds in the CMS lines showed striking similarities with changes that had been previously reported in MADS box genes mutants of *Arabidopsis thaliana*. In floral mutant *ap3* and *pi*, petals were replaced by sepals and stamens were converted into carpel-like structures [26]. These morphological traits indicated that regulation of MADS box of genes and/or other components in the same pathway was disturbed in many CMS lines. Studies in CMS lines of *N. tabacum*, *D. carota*, *T. aestivum* and *B. napus* had proved that, down-regulation of MADS box gene was concordant with floral phenotypes of CMS lines because of misunderstanding of foreign mitochondria [21], [23], [24], [25]. In spite of this progress, mechanism governing functions of nucleus and mitochondria in flower development of CMS line was still unclear. However, hybrid CMS line was indeed an ideal model to analyze correlations that involved in floral bud formation and organ differentiation between nuclear and mitochondrial genomes.

The use of RNA-Seq [27], a high-throughput deep-sequencing technology, has been developed as a novel approach for transcriptome profiling. In recent years, RNA-Seq has been widely applied in transcript profiling of rape [*Brassica napus*], grape [*Vitis vinifera*], tea [*Camellia sinensis*], and sweet orange [*Citrus sinensis*] [28], [29], [30], [31]. Using RNA-Seq analysis, we compared the gene expression profiles of floral buds of G1+HBP that had defects in stamen development to that of HBP; the identified transcripts were compared with the most recently released *C. clementine* genome sequence (http://www.phytozome.net/Clementine) [32]. The identification of CMS related genes provided new insights into interactions between nucleus and mitochondria.

## Materials and Methods

### Plant Materials and RNA Preparation

The male sterile somatic cybrid (G1+HBP) was derived from somatic fusion between embryogenic callus protoplasts of *Citrus unshiu* Marc. cv. Guoqing No. 1 (G1) and mesophyll protoplasts of Hirado Buntan pummelo (*Citrus grandis* (L.) Osbeck) (HBP). Their nuclear and cytoplasmic genome composition was verified [10] and further confirmed [13]. G1+HBP and HBP plants were grafted onto trifoliate orange seedling rootstock at the same time, and planted in the experimental field of the National Citrus Breeding Center at HuaZhong Agricultural University. Floral buds were carefully isolated from HBP and G1+HBP and stored according to their development at the following stages: sepal primordia, petal primordia, stamen primordia, pistil primordia, and full-developed flower. Stages were defined in accordance with paraffin section screening. Floral buds were collected from at least 20 inflorescences on each plant, and from at least two trees of each line, different floral buds were collected at approximately the same time of the day, all samples were mixed as a pool. Parts of them were fixed in FAA, and dehydrated through graded ethanol to 70% for paraffin section preparation. The remaining parts of the floral buds were stored at −80°C for RNA preparation.

Total RNA was extracted according to a published protocol [33]. Firstly, the integrity of the RNA was assessed in a 1% agarose gel, stained with ethidium bromide. Secondly, RNA samples were quantified by Nanodrop ND 1000 spectrophotometer to test protein contamination (A260/A280) and reagent contamination (A230/A280). Finally, the RIN (RNA integrity number) was evaluated by Agilent Technologies 2100 Bioanalyzer, and the value of all samples were greater than 9.4.

### Paraffin Section and RNA *in situ* Hybridization

Paraffin sections were carried out to identify critical stage at which morphology of floral buds in G1+HBP started to deviate. At different developmental stages as described above, the floral buds were fixed overnight in FAA, and then dehydrated through ethanol to 70% for long-term storage. Fixed tissues were continuously dehydrated and embedded in wax, and sections (9 µm) were fixed to lysine treated slides. Specific primers were used to amplify the probe fragments that were cloned into the pGEM-T vector (Promega, USA). Probes were labeled *in vitro* with the digoxigenin-UTP by SP6 or T7 RNA polymerase transcription kit (Roche, Switzerland). *In situ* pre-hybridization, hybridization and detection were performed as previously described [34].

### Scanning Electron Microscopy

Fresh stamens of HBP and G1+HBP were collected at the same time according to their developmental stages and fixed overnight in 2% glutaraldehyde, and dehydrated through graded ethanol. All samples were dried, sputter-coated and analyzed as previously described [35].

### RNA-Seq, Data Processing and Normalization

20 µg total RNA was prepared to enrich mRNA by using the oligo (dT) magnetic beads. After adding fragmentation buffer, the mRNA was interrupted to short fragments (approximately 200 bp). The first strand cDNA was then synthesized with random hexamer-primer using the mRNA fragments as templates. Buffer, dNTPs, RNase H and DNA polymerase I were added to synthesize the second strand [27]. The double-strand cDNA was purified with QiaQuick PCR extraction kit and washed with EB buffer for end repair and single nucleotide A (adenine) addition. Finally, sequencing adaptors were ligated to the fragments. The required fragments was purified by agarose gel electrophoresis and enriched by PCR amplification. The library products were ready for sequencing analysis via Illumina HiSeq™ 2000.

“Clean Reads" were filtered from the raw reads as follows: 1) reliable: removing low-quality reads containing ambiguous nucleotides or adaptor sequences. 2) high-quality: discarding reads in which unknown bases were more than 10% and removing low-quality reads. Gene expression levels were calculated by RPKM method (Reads Per kb per Million reads) [27], if there were more than one transcript for a gene, the longest one was used to calculate its expression level and coverage. The RPKM method was able to eliminate the influence of different gene length and sequencing discrepancy on the calculation of gene expression. Thus, the calculated gene expression could be directly used for comparing the differences in gene expression among samples.

### Alignment and Functional Analysis of Differentially Expressed Genes (DEGs)

Statistical analysis was conducted to summarize the number of clean reads that aligned to the recently released reference genome (http://www.phytozome.net/Clementine) [32] using SOAPaligner/soap2 [36]. Referring to the published results [37], [38], [39], [40], the Poisson model provides a natural framework for identifying differentially expressed genes. Denoted the number of unambiguous clean tags from a given gene as x, given every gene’s expression occupied only a small part of the library, *p*(*x*) would closely follow the Poisson distribution.
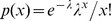
(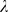 is the real transcripts of the gene). Given every gene’s expression occupied only a small part of the library, P- value that corresponded to DEGs test would closely follow the Poisson distribution. FDR (False Discovery Rate) was a method to determine the threshold of P-value in multiple tests [41]. We used “FDR ≤0.001 and the absolute value of log_2_Ratio ≥1" as the threshold to judge the significance of the difference in gene expression.

The statistical analyses identified DEGs between G1+HBP and HBP at petal primordia and stamen primordia stages. Hierarchical clustering of DEGs was performed using the cluster software and Java Treeview software [42], and GO function analysis was then performed using Blast2Go. GO enrichment analysis provided all GO terms that significantly enriched in DEGs comparing to the genome background, and filtered the DEGs that correspond to biological functions.

### Real-time Quantitative RT-PCR Validation

First-strand cDNA synthesis was performed with 1400 ng of total RNA using RevertAid™ First Strand cDNA Synthesis Kit (Ferment), according to the described protocol. The expression profiles of 22 DEGs were identified by real-time PCR with SYBR green chemistry (QIAGEN, Germany). Gene-specific primers were designed with the Primer Express software (PE applied Biosystems, USA). A primer was also designed for actin gene to normalize the amplification efficiency. Reactions were performed with the SYBR Green PCR Master Mix in the ABI 7500 Real-time system. The output results were analyzed by the instrument on-board software Sequence Detector Version 1.3.1 (PE Applied Biosystems). The real-time PCR was conducted with 4 replicates for each samples, and data were indicated as means ± SE (n = 3).

## Results

### Floral Bud Development in HBP and its Male Sterile Cybrid G1+HBP

**Figure 1:**
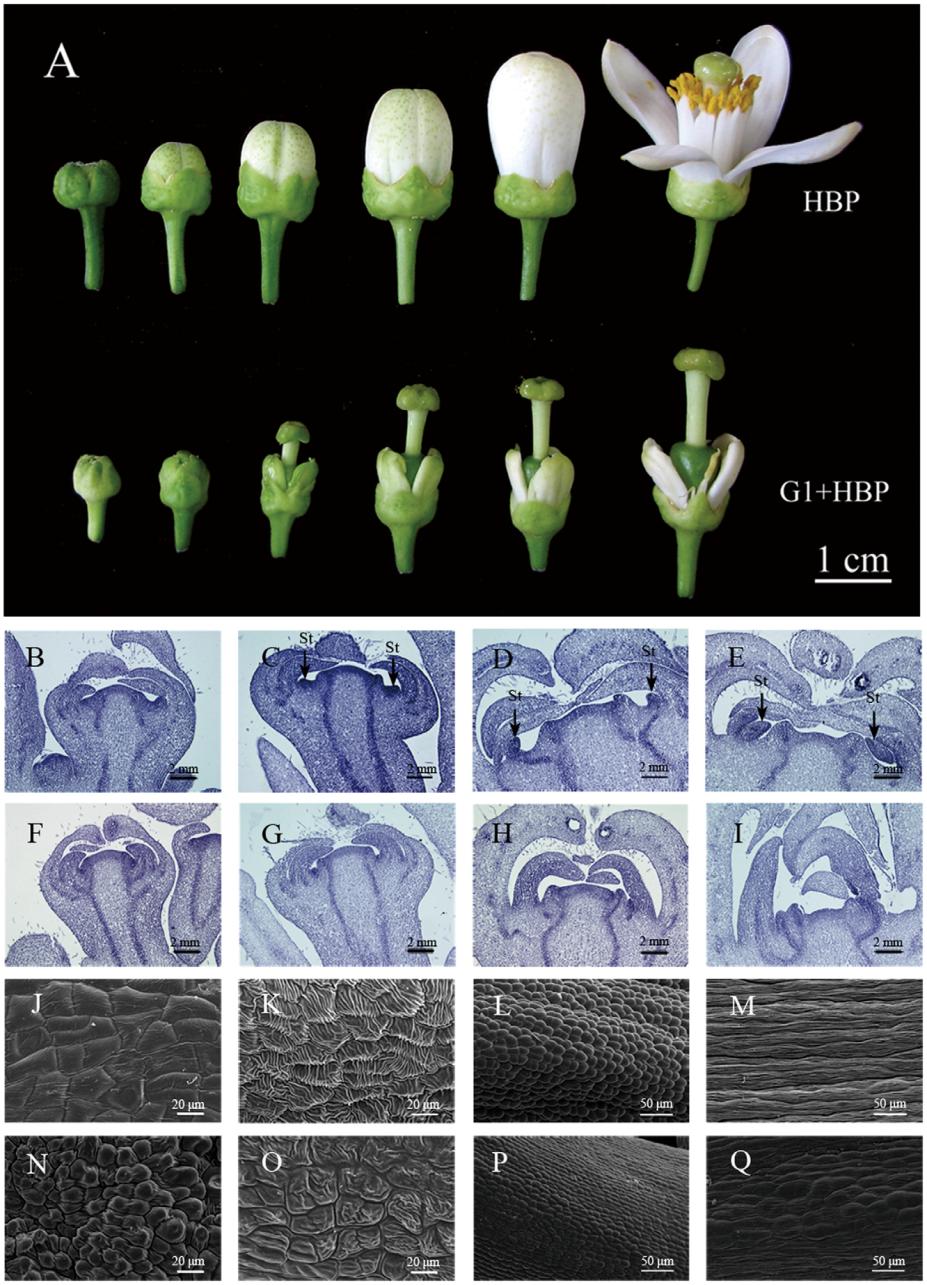
Phenotypic characterization of floral buds development in HBP and G1+HBP. Phenotype of floral buds during different developmental stages [A]. Paraffin section analysis of HBP [B–E] and G1+HBP [F–I]. Petal developmental stage [B, F]. Stamen primordia initiating stage [C, G]. Stamen developmental stage [D, H]. Stamen mature stage [E, I]. Scanning electron microscopy analysis of stamens in HBP [J–M] and G1+HBP [N–Q]. Surface cells of anthers of HBP [J, K] and G1+HBP [N, O]. Surface cells of filaments of HBP [L, M] and G1+HBP [P, Q]. Stamens obtained from stamen developmental stage [J, L, N, P]. Stamens obtained from stamen mature stage [K, M, O, Q].

As different developmental stages of floral buds were shown in Figure 1A, floral buds of G1+HBP were HBP-like in terms of sepal morphology and pistil character, and the spatial distribution of floral organs was similar to that of HBP, proving its consistent nuclear inheritance. Histological sections revealed that floral bud morphology of G1+HBP and HBP remained unchanged until stamen primordia emerged (Figure 1B, F). However, at stamen primordia stage, a furrow that divided floral apical meristem region and petal primordia became broader and protruded stamen primordia was discernible, phenotypic differences between flowers of G1+HBP and HBP were clearly distinguishable (Figure 1C–E, G–I). The floral buds of G1+HBP indicated no/less differentiation of stamen primordia structures (Figure 1D, H). During the fully developed stage, when all flower organs were established and final shaped stamen in HBP reached to the stigma, G1+HBP displayed reduced petals in size and width, which resulted in corolla split (Figure 1A). Unmodified stamens of mature flower in HBP differentiated into filaments and anthers; however, mature flowers of G1+HBP showed few aberrant stamens fused to petals/carpel without typical filaments and anthers (Figure 1A).

Scanning electron microscope analysis of HBP anther surface and filament surface revealed that the anther surface cells were polygonal in shape (Figure 1J) and showed pinched texture when mature anthers entered a dehiscence program that led to pollen release (Figure 1K). Filament surface of developed stamen showed rounded cells (Figure 1L), and the cells were uniform in shape and size with longitudinal spindle shape at the anther mature stage (Figure 1M). However, in G1+HBP, anther-like surface cells were dome shaped with undivided cells and the pinched texture of them was distinguishable from that of HBP (Figure 1J, K, N, O), surface cells of filament-like structures were disorganized and divided into two types of cells; one type was typical to that of HBP, whereas the other type exhibited bubble-like protrusions (Figure 1P, Q). The results indicated that G1+HBP displayed floral alterations just in the second and third whorls, and anther-like structures in G1+HBP might be dysfunctional and unable to split and release pollen. All of these floral bud characteristics suggested that G1+HBP was completely male sterile, even though with a few stamen-like structures.

### Illumina Sequencing and Mapping of the Reference Genome

As the morphology of young floral buds of G1+HBP were similar to that of HBP, we reasoned that analysis of transcript profiles of early stage floral buds would allow us to trace the initiation of gene expression alterations that led to abnormal flower morphology changes of G1+HBP. To characterize changes in gene expression between G1+HBP and HBP, floral buds at stages of petal primordia and stamen primordia were collected from these two lines. Four pools of mRNA samples, one representing each stage of one line, were used to build libraries for high-throughput parallel RNA-Seq. Each stage of lines was represented by at approximately 12 million reads, a total of 49,114,530 successful sequences were obtained (Table 1), each 49-bp in length. Sequence saturation analysis indicated that with the number of reads increasing, the growth rate of detected genes tended towards saturation. When the number of reads in this study reached 2.5 million, the growth rate of detected transcripts became flattened (Figure S1). Correlation efficient of the raw data revealed high repeatability between two stages of HBP and G1+HBP; both species had a value of r≈0.98 (Figure S2) indicating high reproducible levels of RNA-Seq technology. Gene coverage meant the percentage of a gene covered by reads, and the value was equal to the ratio of the bases number in a gene covered by unique mapping reads to the total bases number of that gene. In this study, gene coverage of each library was similar to the others, which varied greatly from 0.49% to 99.98% (Figure S3). All of the sequencing assessments indicated that the reads density was sufficient for quantitative analysis of gene expression.

**Table 1:** Summary of RNA-Seq read number in the fertile pummelo (HBP) and male sterile cybrid pummelo (G1+HBP).

	HBP-Pe	HBP-St	G1+HBP-Pe	G1+HBP-St
**Total reads collected**	12,109,778 (100%)	12,627,744 (100%)	11,750,250 (100%)	12,626,758 (100%)
**Low-quality reads**	125,653 (1.04%)	124,265 (0.98%)	117,971 (1.00%)	152,959 (1.21%)
**High-quality reads**	11,984,125 (98.96%)	12,503,479 (99.02%)	11,632,279 (99.00%)	12,473,799 (98.79%)
**Reads mapped to genome**	9,386,751 (77.51%)	9,770,917 (77.38%)	9,117,711 (77.60%)	9,785,580 (77.50%)
**Unique-match**	8,889,024 (73.40%)	9,272,996 (73.43%)	8,633,593 (73.48%)	9,275,930 (73.46%)
**Multi-position match**	497,727 (4.11%)	497,921 (3.94%)	484,118 (4.12%)	509,650 (4.04%)
**Unmapped reads**	2,597,374 (21.45%)	2,732,562 (21.64%)	2,514,568 (21.40%)	2,688,219 (21.29%)
**Unique-match to genes**	4,614,350 (38.10%)	4,846,959 (38.38%)	4,521,048 (38.48%)	4,857,958 (38.47%)

Of the total reads, more than 98% were defined as high-quality by removing low-quality with ambiguous nucleotides and adaptor sequence (Table 1). The high-quality sequences were aligned to the recently released *C*. *clementine* reference genome (http://www.phytozome.net/Clementine) [32] allowing for two bases mismatch. Of the high-quality reads, more than 78% matched either to a unique or multiple genomic locations (Table 1). However, only the reads which uniquely mapped to the reference genome were used in gene expression analysis of all libraries. Among the uniquely mapped reads, 4,614,350, 4,846,959, 4,521,048 and 4,857,958 were mapped to reference genes in each stage of HBP and G1+HBP (Table 1).

### Global Analysis and Functional Classification of DEGs

One of the primary goals of the transcriptome study was to perform global analysis of transcriptive variation in different libraries. In this study, during petal primordia stage, only 54 genes exhibited up- or down-regulation between HBP and G1+HBP (Table S1). The number of DEGs increased with progressing developmental stages. Following the same filtering principles, 247 genes were identified with up- or down-regulation in G1+HBP compared with HBP at stamen primordia stage (Table S1), 41 of them showed the same trend of up- or down-regulation at different levels as those at petal primordia stage.

**Figure 2:**
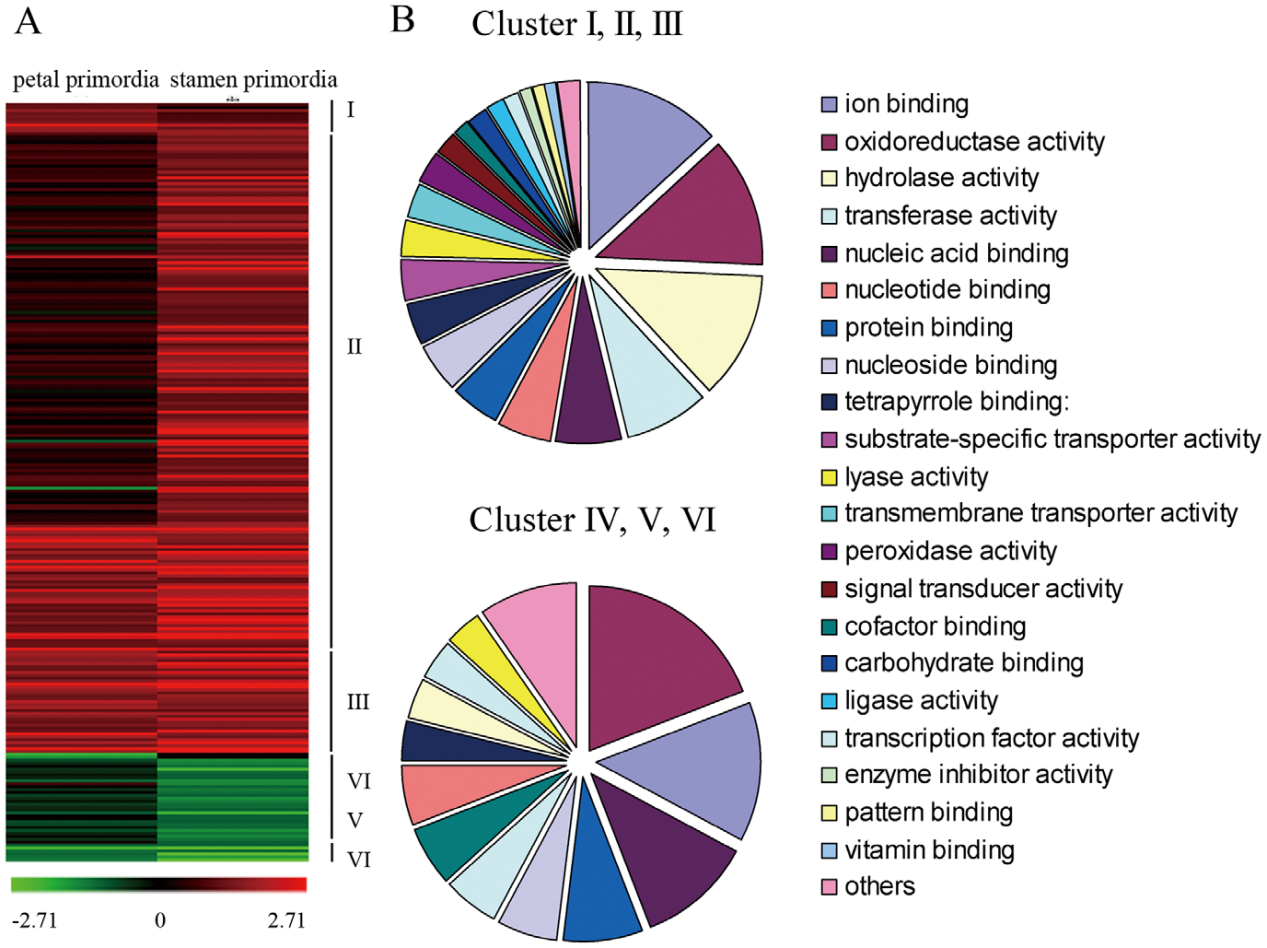
Hierarchical cluster analyses and functional categories of differentially expressed genes in the experiment. Hierarchical cluster analyses [A], genes down-regulated in G1+HBP compared to HBP were depicted in green, and up-regulated genes were depicted in red. The diagram was generated using log_2_-transformed ratio values. Functional categories within up- and down-regulated genes in G1+HBP compared to HBP [B]. The set of the 261 genes were significantly enriched in genes involved in ion binding and oxidoreductase activity.

Cluster analysis of the DEGs was performed in six clusters with the correlated expression profile (Figure 2, Table S2). Genes in clusters I and II contained genes with higher expression in G1+HBP compared with HBP at petal primordia stage and stamen primordia stage, respectively. In contrast, clusters IV, V revealed significantly reduced transcripts in G1+HBP at each floral developmental stage. Clusters III and VI consisted of genes which showed activated and reduced expression in the floral buds of G1+HBP compared with that of HBP in the two stages. Within the clusters of co-expressed genes, we found an enrichment of several known or predicted functions during floral bud development. In particular, 25 of the 177 genes in cluster II (Table S2) were specifically or predominantly expressed during stages of floral meristem or organ development. Genes correlated with floral bud development and male sterility, were repressed in G1+HBP. These genes included a floral meristem identity gene (*LFY*), a gene encoded PPR motif protein in cluster V and a gene (*NDB4*) located on the mitochondria inner membrane in cluster VI. Other genes closely related with hormone synthesis and metabolism also showed differentially expressed profiles, for examples, a gene encoding ACC synthase protein (*ACS1*), auxin-responsive genes (*DFL1, GH3, SAUR-like*) and gibberellin-regulation gene (*GA20-OX1*). Additionally, a number of transcription factors (including MYB and NAC) were identified. The statistical analysis data demonstrated that DEGs identified in this experiment were likely to be involved in floral bud development and hormone metabolism, and that most of the observed genes might contribute to abnormal floral development in G1+HBP.

To gain insight into the functional categories which were altered between G1+HBP and HBP, groups of up- and down- regulated genes were annotated according to function annotation convention. Gene ontology (GO) categories were assigned to significant DEGs based on the TAIR GO slim provided by blast2GO. The GO terms consisted of following three ontologies: molecular function, cellular component and biological process. Based on the molecular functional categories, genes involved in ion binding, oxidoreductase activity and nucleic acid binding were enriched among genes in groups of up-regulated and down-regulated in G1+HBP (Figure 2). Moreover, up-regulated genes were enriched in the categories of peroxidase activity and signal transducer activity. The majority of up-regulated genes seemed to be related to four major biological changes, including metabolic process, response to stress, biosynthetic process, as well as oxidation reduction, whereas down-regulated genes were mainly involved in cellular metabolic process, regulation of biological process and oxidation reduction (Figure S4).

### Verification on Expression Patterns of the CMS-related Genes

**Figure 3:**
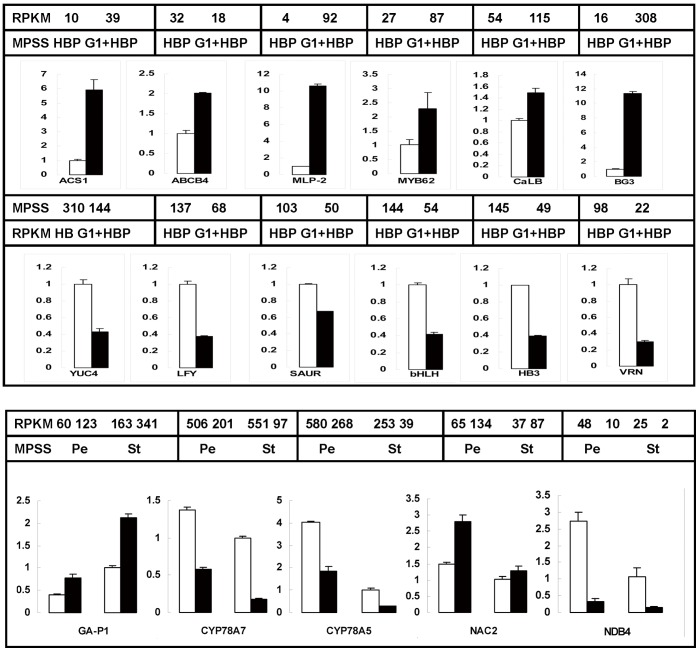
Real-time PCR confirmation of the differentially expressed genes between HBP (white columns) and G1+HBP (black columns). Columns and bars represented the means and standard error (n = 3) respectively. The transcript abundance from RNA-seq was added on the top of each gene. RPKM, reads per kb per million reads. Pe, petal primodia initiating stage. St, stamen primodia initiating stage.

**Figure 4:**
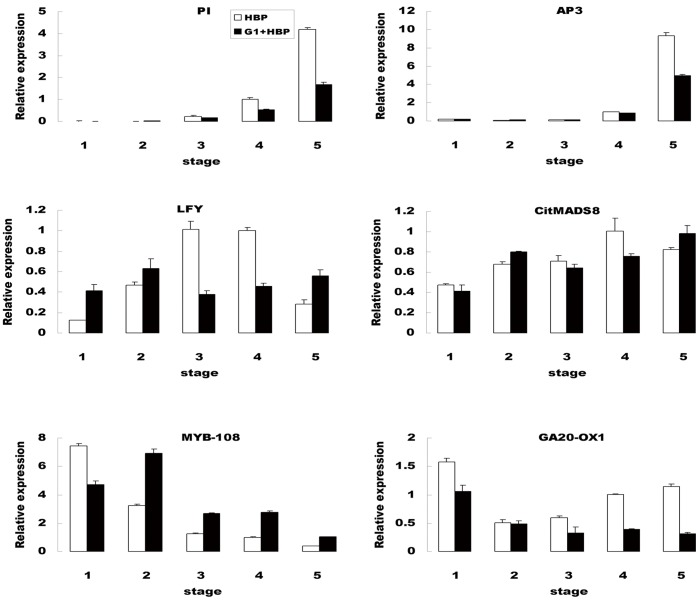
Transcript level of 6 selected genes at different stages of floral buds development in HBP (white columns) and G1+HBP (black columns). Stage 1, undetermined lateral bud. Stage 2, sepal primordia initiating. Stage 3, sepal development and petal primordia initiating. Stage 4, petal development and stamen primordia initiating. Stage 5, stamen development and pistil primordia initiating. Relative transcript levels were calculated by real-time PCR with actin as a standard. Data are means ± SE of three separate measurements.

To confirm that the unique-match genes from the deep sequencing and bioinformatics analysis were indeed differentially expressed, a total of 22 genes, including 19 significant DEGs and three MADS box genes (*PI, AP3, citMADS8*) which were also detected by RNA-Seq in this analysis, were selected to design gene-specific primers (Table S3) for real-time PCR analysis. The relative transcript levels of HBP and G1+HBP were compared with those of RNA-Seq data. Despite some quantitative differences in expression level, real-time PCR results revealed the same expression tendency as RNA-Seq data. Figure 3 showed the expression levels of 17 genes (8 for induced, 9 for repressed) in HBP and G1+HBP. As analysed by real-time PCR, it was attractive that expression level of a gene encoding ACC synthase showed 5.8 times up-regulation in G1+HBP than in HBP, consistent with RNA-Seq data that the ratio of the gene expression level in G1+HBP to that in HBP was 3.9 to 1. Furthermore, the expression profile of 6 genes, including 4 citrus putative homologs for floral integrator or organ identity genes (*LFY, PI, AP3, citMADS8*), and 2 genes involved in regulation pathway of flower development (*MYB-108, GA20-OX1*) were analyzed at 5 stages during flower development between HBP and G1+HBP (Figure 4). As expected, expression of *PI* was detected at stamen primordia initiating stage and gradually showed significant down-regulation in floral buds of G1+HBP at stages of stamen development and pistil primordia initiation (Figure 4). The reported citrus *AP3* homolog gene (*citMADS8*) showed no significant expression profile between G1+HBP and HBP (Figure 4). However, expression of another citrus *AP3* transcript based on the RNA-Seq was coincided with the expression profile of *PI*, indicating that the retarded stamen differentiation and development were partially attributable to repressed expression of MADS box B-function genes. It was noticeable that expression of *MYB 108* (a gene involved in stamen maturation and filament elongation) was up-regulated throughout stages of floral bud development in G1+HBP (Figure 4).

### *In situ* Localization of *AP3* and *PI* Transcripts in Floral Buds

**Figure 5:**
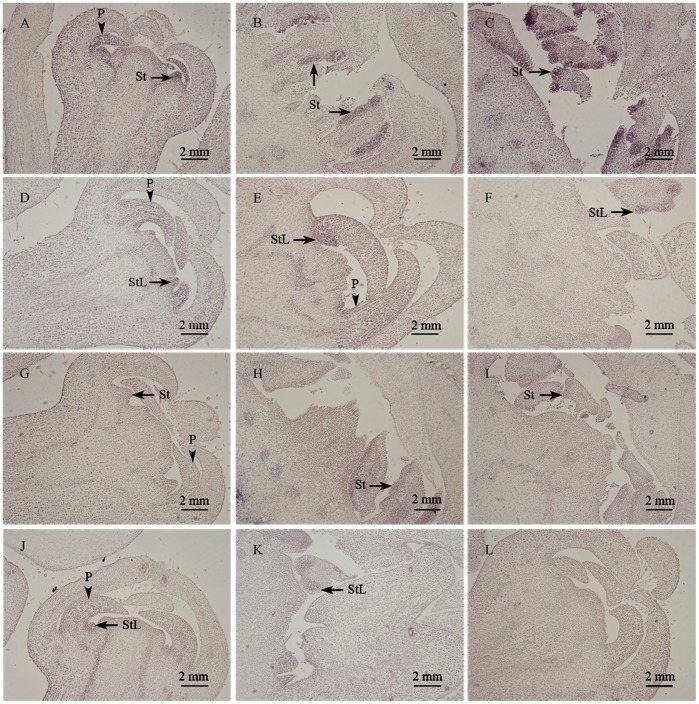
*In situ* hybridization patterns of *PISTILLATA* (*PI*) and *APETALA* (*AP3*) transcripts in HBP and G1+HBP floral buds. Bright-field micrographs show longitudinal sections of HBP [A–C, G–I] and of G1+HBP [D–F, J–L] floral buds. Hybridization signals appear as dark brown-purple color. Sections in [A–F] were hybridized with *PI* anti-sense probe. *PI* expression was detected in petal and stamen-like structures in G1+HBP [D–F] in a pattern similar to that found in corresponding HBP floral buds [A–C]. Micrographs in [G–L] showed sections of HBP [G–I] and G1+HBP [J–K] floral buds hybridized with an *AP3* anti-sense probe. Hybridization with an *AP3* sense probe did not yield any signal above background [L]. Arrow head, P (petal). Arrow, St (stamen), StL (stamen-like structure).

To determine whether down-regulation of *AP3* and *PI* expression in floral buds of G1+HBP were a consequence of either reduced expression levels or reduced expression domains, or both, we analyzed in detail the expression profile of *AP3* and *PI* during stages of floral bud development. In HBP, *PI* expression was uniformly detected in petal and stamen primordia (Figure 5A). During early stages of stamen differentiation and development, *PI* transcripts accumulated in the filament and the anther wall (Figure 5B). At the stamen mature stage, it seemed to be preferentially expressed in the microspore mother cells (Figure 5C). In G1+HBP, *PI* transcript was detected at petal primordia and stamen-like primordia structures, as well as that of HBP (Figure 5D, E). However, in the stamen priomordia stages, *PI* expression was more uniform in the abnormal stamen structure, and weak expression was detected in the microspore mother cells (Figure 5E, F). In corresponding hybridization with an *AP3* anti-sense probe, we detected transcripts in petal and stamen primordia as well as the shaped filament and anther of HBP (Figure 5G–K), in a pattern that was similar to that of *PI.* For negative control, hybridization with a sense probe showed only the background signal (Figure 5L). The results described above coincided with real-time PCR results, which indicated that the transcription levels of *AP3* and *PI* were higher in the stamen development stage than they were in stamen primordia stage (Figure 4, Figure 5A–K). Thus, *AP3* and *PI* genes were not only involved in specifying stamen and petal primordia identity but also played important role in the developmental process of these organs. Furthermore, in G1+HBP, *AP3* and *PI* showed reduced expression levels but no ectopic expression profiles. These results confirmed the temporal gene expression results, suggesting that citrus class-B MADS-box genes could be closely associated with the reduced petal and stamen development in G1+HBP.

## Discussion

In *Citrus* genus, Satsuma mandarin was verified to be CMS type [11]. But because of its male sterile, nucellar polyembryonic traits and lacking corresponding wild male fertile type, gene exploitation from Satsuma mandarin was difficult. In this study, the novel male sterile line (G1+HBP) containing the nuclear genome of HBP combined with mitochondrial genome from Satsuma mandarin [10], [13], showed a resemblance to the homeotic mutant phenotypes (Figure1A). We demonstrated that no deviations from normal flower development were observed during initial steps of floral bud in G1+HBP, and that organ morphological differences appeared when stamens started to differentiate (Figure 1B–I). These results agreed with the phenomenon that had been described in homeotic CMS lines of *T. aestivum*, *D. carota*, and *B. napus* [21], [23], [24], [25]. These phenotypic aberrations suggested that foreign mitochondrial genome in G1+HBP led at least in part to retrograde signaling that changed expressions of nuclear genes and resulted in male sterility. Because of the dynamic morphological changes, the expression levels of a large number of genes involved in a variety of developmental aspects, such as floral meristem transformation and floral organ differentiation, were expected to change dramatically at critical stages of stamen development. Wished to understand, in as much detail as possible, how foreign mitochondrial genome regulated nuclear gene expression in male sterility line (G1+HBP) in this analysis, RNA-Seq analysis of nuclear gene expression profiles of floral buds between G1+HBP and HBP was performed. RNA-Seq provided a far more precise measurement of levels of transcripts for the resulting sequence reads were individually mapped to the reference genome and normalized to RPKM at gene expression level [27], [40]. RNA-Seq was highly accurate for quantifying expression levels, and showed low (if any) background signal and high levels of reproducibility for both technical and biological replicates [27], [38], [39], [40]. It was necessary to perform biological replicates, but we tried to avoid variation for all samples were mixed as a pool for RNA-Seq analysis. Taking high levels of reproducibility of RNA-Seq and experiment consumption into consideration, we just performed one Illumina run for each sample pool. In this study, correlation efficient of the raw data between two stages of HBP and G1+HBP revealed high repeatability (Figure S2), which confirmed that the results obtained from RNA-Seq in this analysis were reliable.

### Transcriptional Regulation of Floral Bud Development in Male Sterile Cybrid G1+HBP

In this study, we successfully identified 54 (46 for induced, 8 for repressed) and 247 (212 for induced and 35 for repressed) DEGs at 0.05% significance level that were changed at least two fold during petal primordia and stamen primordia stages in G1+HBP compared with HBP (Table S1). Most of these genes were involved in early stages of floral identification, such as floral meristem transformation or establishment of the floral meristem into different types of floral organs. Among them, 39 were previously reported as being involved in early flower development in *Arabidopsis* [43] (Table S2). Hierarchical clustering of the DEGs according to their co-expressed profiles indicated a transcriptional cascade, in which relatively few genes were activated at early stage, more genes were activated or repressed as the developmental progress proceeded and a specific set of genes were activated in the development of floral organs (Figure 2). The global transcript profile analysis provided a comprehensive structure with each gene represented by its absolute expression level during the stages of floral bud development. A number of genes and transcription factors with known function were identified in these DEGs, e.g. homolog genes for floral organ differentiation (*LFY/PI*) and other CMS-related genes (*YUC/GA20-OX1/MYB108*) (Figures 3, 4) whose functions were clearly linked with the morphological changes of aberrant flowers. Analysis of the datasets derived from this experiment led to identify genes that were likely involved in the control of key developmental processes during floral bud development.

To further characterize the DEGs in this analysis, GO prediction was performed using Blast2Go. We found that none of biological processes were significantly enriched in the dataset when compared to their distribution of all differently expressed genes. However, when the up- and down-regulated genes were taken into consideration, we found that up-regulated genes mainly involved in categories of metabolic process and response to stress, whereas cellular metabolic process, regulation of biological process and oxidation reduction were enriched in down-regulated genes (Figure S4). When compared with HBP, the genes involved in responses to stress (mainly MYB, NAC transcription factors) were significantly up-regulated in G1+HBP at the critical stage of flower development (Figures 3, 4). Both MYB and NAC were categories of transcription factors well known to control multiple processes in plants, including not only responses to hormones and biotic stress but also flower development and reproduction [44]. As reported in a previous study [13], no particular stresses were observed for G1+HBP. The up-regulation of stress responsive transcription factors could be attributed to the foreign mitochondria, which coincided with the results of another citrus cybrid between *C. reticulata* and *C. limon* [45]. Normal reproductive floral organ development was essential for plant propagation, genetic buffering by activation of stress-related transcription factors might be beneficial for the CMS line to overcome reproductive developmental obstacles to survive.

One hypothesis for abnormal floral phenotype in CMS line was that stamen development required effective mitochondria activity that could not be provided by foreign mitochondria during the critical developmental stage (Hanson and Bentolila, 2004). Plant mitochondria contained two respiratory electron transport chains, with pathways transferring electron from NADPH to O_2_ with or without generating energy carrier ATP [46], [47]. The responses of mitochondrial electron transport were mediated in part by retrograded signaling between nucleus and mitochondria. Genes encoding PPR motif domain and NAD(P)H dehydrogenase (*NDB4*) were involved in energy and non-energy electron transport chains, respectively. Moreover, multiple PPR protein encoding genes which involved in plastid RNA editing and translation were linked with many of the known male fertility restorer loci [46], [47]. In this study, we found that expression levels of the two genes were significantly repressed in G1+HBP (Figure 3). The results suggested that mitochondria activity might be reduced in G1+HBP and partially contributed to male sterile characteristics.

### Identification of Potential Genes and Metabolism Pathways Involved in Floral Bud Initiation and Development in Male Sterile Cybrid G1+HBP

Flowers of G1+HBP displayed morphological changes that partially resembled that of the *A. thaliana* MADS box mutants of *AP3* and *PI*, which were involved in the speciation of petals and stamens. Several published studies indicated that orthologues of floral organ differentiation genes were affected in many CMS plants, and that, because of their whorl specific effects in flower development, the floral organ identity genes likely constituted ‘perfect targets’ for CMS retrograde signaling. In fact, repressed expression of *AP3* and *PI* in the flowers of the CMS line had been previously demonstrated in other species, such as brassica and wheat [21], [25], [48]. Though no significant expression differences of *AP3* and *PI* were detected in RNA-Seq data due to floral buds at early developmental stages might be collected, and transcripts were diluted in RNA samples. Results of the present RNA-Seq analysis suggested that differences in *AP3* and *PI* between G1+HBP and HBP were more pronounced at the later stage of floral bud development. Results of real-time PCR was in accordance with that of RNA-Seq, compared with that in HBP, expression levels of *AP3* and *PI* in G1+HBP were not repressed at sepal initiation stage but were down-regulated when stamen primordia initiated (Figure 4).

During floral speciation in *A. thaliana*, expression of *AP3* and *PI* could be directly or indirectly induced by combined activity of the floral meristem identity genes *APETALA1* (*AP1*) and *LEAFY* (*LFY*) [49], [50]. After flower development started, AP3/PI transcription factor complex was regulated by an anti-regulatory feedback loop, *AP3* and *PI* functioned with each other to balance the level of the transcription factor complex. Thus, disruption of one of the two genes resulted in a loss of function of the regulatory complex [51], [52]. Studies on other species (tobacco, wheat, carrot, *Brassica*) indicated that down-regulation of *AP3* and *PI* followed with homeotic conversion of the third-whorl organs into fourth-whorl like structures [22], [23], [24], [25]. In this study, down-regulation of *PI* and *AP3* in G1+HBP consequently reduced the activity of the AP3/PI transcription factor dimer complex, which might be resulted in reduced petals and retarded stamen primordia development but no significant floral organ homeotic conversions. *In situ* hybridization analysis revealed that expression levels of *AP3* and *PI* were weak in the abnormal stamen-like structures (Figure 5D–F, J–K), which most likely prevented the homeotic conversion of stamens into pistil. Similar phenomenon had been described in wheat CMS line, which indicated that stamens showed carpel-like structures, yet still accumulated weak *PI* transcripts [48]. However, in CMS lines of *Brassica* [21], *AP3* and *PI* transcripts were restricted to petals and not detectable in the third-whorl organs. Thus the floral morphology was typical as that of the *A. thaliana* MADS box mutants of *AP3* and *PI*. Taking the morphological changes and gene expression profiles into consideration, it was reasonable to believe that different thresholds of AP3/PI transcription factor activity levels were needed for normal stamen identification and floral organ homeotic conversion. These results indicated that repression of class-B MADS-box genes contributed at least in part to the reduced petal and abnormal stamen development in G1+HBP.

We searched the dataset for DEGs in G1+HBP and identified a number of genes that encoded proteins with known roles in the biosynthesis, or in response to plant hormones auxin and gibberellin (GA) were mis-regulated (Table S1). Both had been previously implicated in regulating distinct processes during flower development [53]. GA had been proved to regulate flower organ development by antagonizing the function of DELLA proteins and partially activating the expression of floral homeotic genes *AP3, PI* [54], [55]. Although it was not demonstrated to be involved in floral organ differentiation, GA was essential for the normal growth and development of these organs [56]. It had been demonstrated that active GA levels were mainly regulated by the expression of GA20-ox genes, which encoding the key enzyme that catalyzed the final step in the biosynthesis pathway of bioactive GAs [56], [57]. Studies on the role of GA in the floral initiation of woody perennial were vast and inconsistent; in citrus, regulation of GA levels in floral bud development was a complex network that involved in both positive and negative processes, and a low level of endogenous GA was correlated with floral initiation [58]. Though the role of GA in floral initiation might be species-specific, its function in the floral organ development was far more general and most likely universal [56]. As that in Arabidopsis, active GA (GA_1_ in citrus) was believed to participate in regulation of vegetative growth and floral bud formation. The genes encoding GA 20-oxidase had also been cloned, and their expression levels were correlated with active GA concentration and elongation in higher plant species [59], [60]. Both RNA-Seq and real-time PCR results indicated that expression level of *GA 20-oxidase* was repressed in G1+HBP. During the initiation stage of floral bud development, the reproductive organs displayed a relatively lower expression level of *GA 20-oxidase* compared with that in vegetative shoots. The gene expression was then gradually up-regulated which coincided with the steps of floral bud development. Those data supported the idea that low level of endogenous GA was needed for floral initiation in citrus [58], [61]. As demonstrated in other species, active levels of GA might be at least partially accounted for normal stamen formation and floral organ size in citrus [62], [63].

Previous studies demonstrated that GAs acted through jasmonate (JA) to promote expression of R2R3 MYB transcription factor needed for stamen development [64], [65]. It was worthy to notice that, when compared with HBP, G1+HBP displayed up-regulation profiles for members of MYB transcription factor (Figures 3, 4). Solid evidences had demonstrated that suitable expression of R2R3 MYB transcription factors (*MYB108, MYB21, MYB24*) was important for stamen development and maturation; however, excessive expression of these genes might attenuate stamen development [54], [65], [66]. As indicated in Figure 4, expression level of *MYB108* (a member of R2R3-MYB transcription factor) was higher in G1+HBP than that in HBP. We hypothesized that *MYB108* might be an important transcription factor for regulating stamen development in HBP; the activated expression profile in G1+HBP might be contributed to retarded stamen development. Suitable gene expression was thought to serve as a genetic buffering mechanism to maintain floral organ function and size throughout developmental stages.

### Conclusions

In summary, our analysis specified the critical stage at which morphology of floral buds in G1+HBP started to deviate and provided a global architecture of the nuclear gene expression changes in the sterile line G1+HBP compared with HBP. Furthermore, we identified that *AP3* and *PI* transcripts, which encoded key transcription factors for stamen identification, were repressed in G1+HBP during stages of floral bud development though they were restricted to correct floral whorls. This study provided new insights and enhanced that nuclear genes regulating floral development constituted ‘perfect targets’ for retrograde signaling between mitochondrial and nuclear genomes.

## Supporting Information

Figure S1 Saturation evaluation of the RNA-seq tags in the four libraries (HBP and G1+HBP at two selected floral bud developmental stages) against sequencing depth. With the number of reads increasing, the number of detected genes was increasing in four libraries. When the number of the reads reached 2.5×10^6^, the growth rate of detected genes became flatten. Pe, petal primordia initiating stage. St, stamen primordia initiating stage.
(TIF)

Figure S2 Correlation analysis of the raw data between two stages of HBP and G1+HBP. The Pearson r values of HBP and G1+HBP were both nearly 0.98. Pe, petal primordia initiating stage. St, stamen primordia initiating stage.
(TIF)

Figure S3 Distribution of gene coverage in HBP and G1+HBP at two stages analyzed. Gene coverage of each library was similar to the others, which varied greatly from 0.49% to 99.98%. Pe, petal primordia initiating stage. St, stamen primordia initiating stage.
(TIF)

Figure S4 Biological process of genes with significantly differential expression profiles between G1+HBP and HBP. Up-regulated genes [A]. Down-regulated genes [B].
(TIF)

Table S1 Listing of differentially expressed genes at petal primordia initiating stage and stamen primordia initiating stage during floral bud development, along with two fold change, and functional categories. A. List of differently expressed genes at petal primordia initiating stage. B. List of differently expressed genes at primordia initiating stage.
(XLS)

Table S2 The 261 genes differentially expressed between G1+HBP and HBP sorted within clusters. Genes in clusters I and II contained genes with higher expression in G1+HBP compared with HBP at petal and stamen primordia initiating stage, respectively. In contrast, clusters IV, V revealed significantly reduced transcripts in G1+HBP at each floral development stage. Clusters III and VI were composed of genes which showed activated and reduced expression in the floral buds of G1+HBP compared with that of HBP in two analyzed stages. For each gene the relative expression value was presented as a log2-value. Only significant values were presented. A positive value indicated that the expression was higher in G1+HBP in comparison to HBP and a negative value indicated a lower expression in G1+HBP in comparison to HBP.
(XLS)

Table S3 Primer sequences for Real-time PCR and probe of ISH.
(XLS)
