# Supplementary figures and images for: Comparative Transcript Profiling of a Male Sterile Cybrid Pummelo and Its Fertile Type Revealed Altered Gene Expression Related to Flower Development
Source: PLoS One. 2012 Aug 28;7(8):e43758. doi: 10.1371/journal.pone.0043758 (PMC3429507; doi:10.1371/journal.pone.0043758)

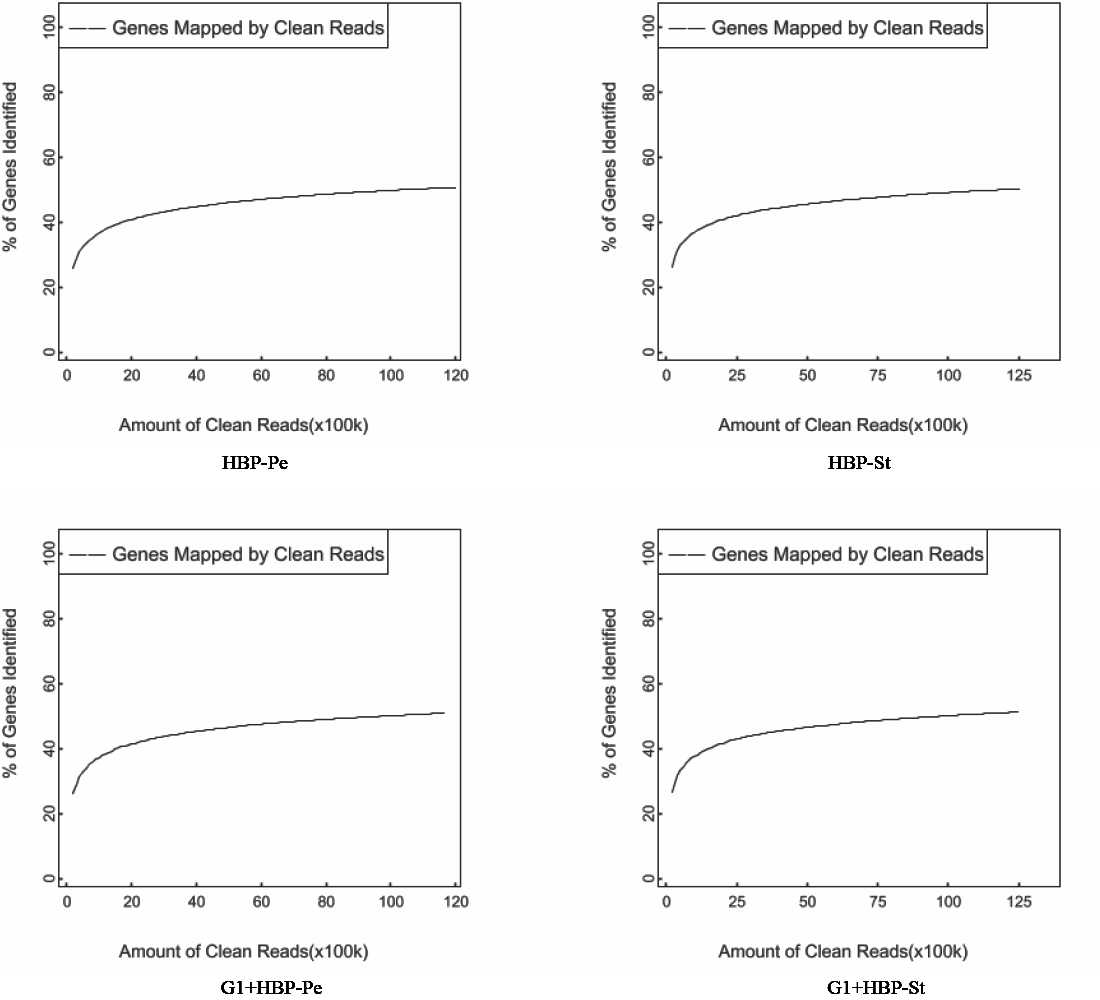

Supplement: Figure S1 — Saturation evaluation of the RNA-seq tags in the four libraries (HBP and G1+HBP at two selected floral bud developmental stages) against sequencing depth. With the number of reads increasing, the number of detected genes was increasing in four libraries. When the number of the reads reached 2.5×10^6^, the growth rate of detected genes became flatten. Pe, petal primordia initiating stage. St, stamen primordia initiating stage. (TIF) [file pone.0043758.s001.tif]

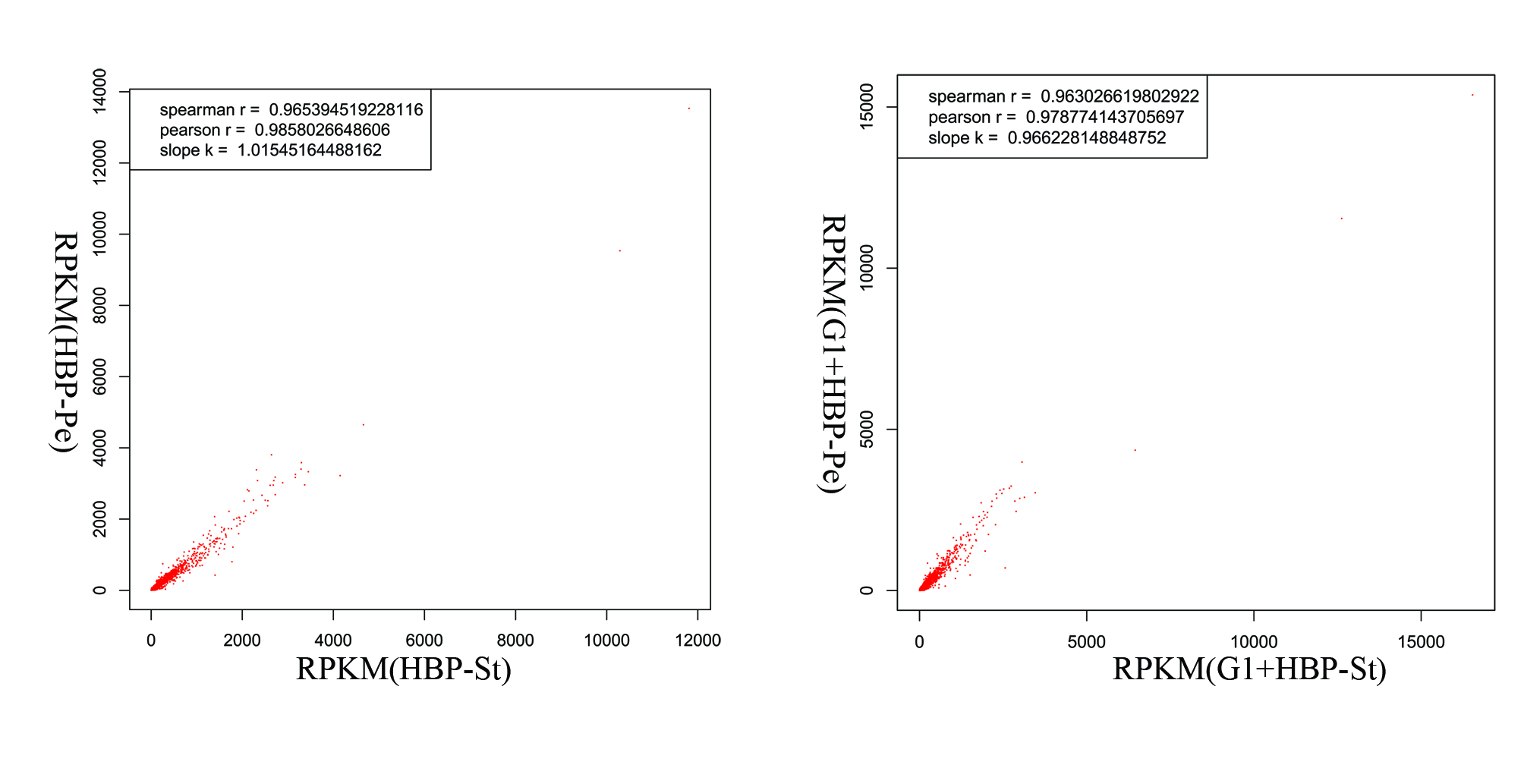

Supplement: Figure S2 — Correlation analysis of the raw data between two stages of HBP and G1+HBP. The Pearson r values of HBP and G1+HBP were both nearly 0.98. Pe, petal primordia initiating stage. St, stamen primordia initiating stage. (TIF) [file pone.0043758.s002.tif]

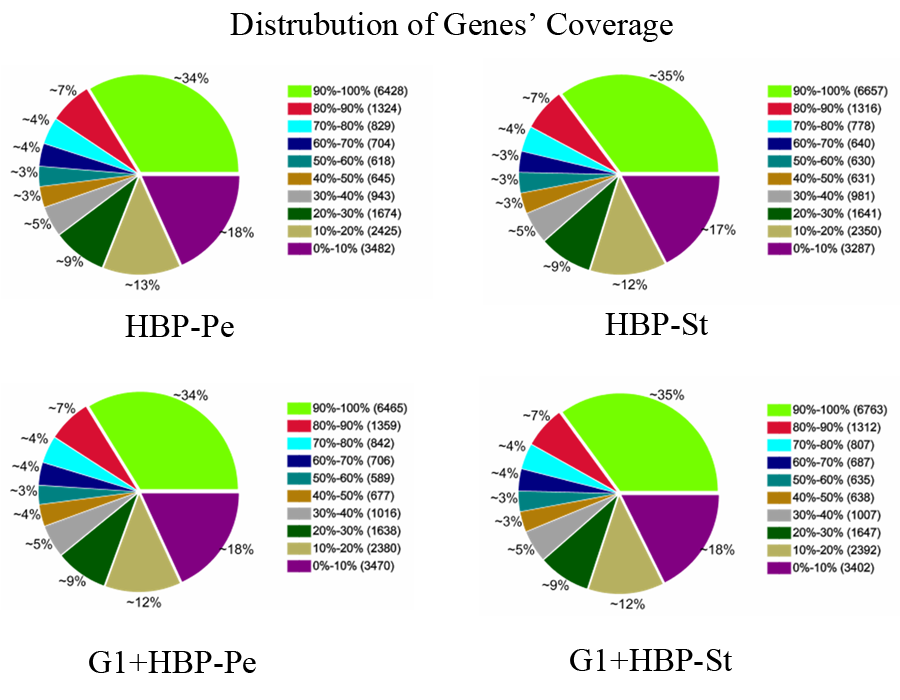

Supplement: Figure S3 — Distribution of gene coverage in HBP and G1+HBP at two stages analyzed. Gene coverage of each library was similar to the others, which varied greatly from 0.49% to 99.98%. Pe, petal primordia initiating stage. St, stamen primordia initiating stage. (TIF) [file pone.0043758.s003.tif]

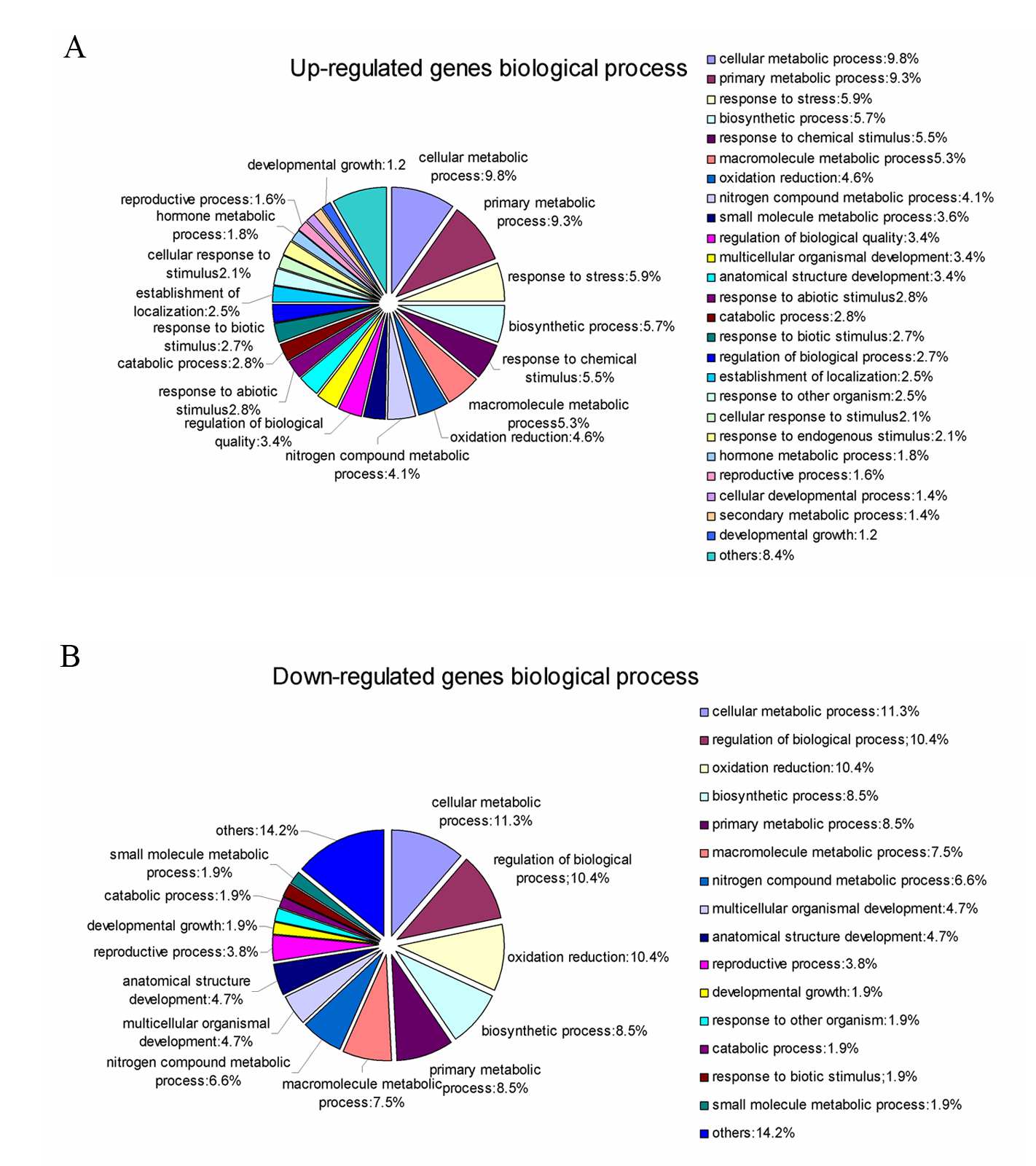

Supplement: Figure S4 — Biological process of genes with significantly differential expression profiles between G1+HBP and HBP. Up-regulated genes [A]. Down-regulated genes [B]. (TIF) [file pone.0043758.s004.tif]
